# From Simulation to Semi-Physical Validation: An Intelligent Jammer-Assisted Radar Anti-Jamming Evolution Method

**DOI:** 10.3390/s26154688

**Published:** 2026-07-23

**Authors:** Feng Xie, Huanyu Liu, Jiaming Wang, Boxu Pei, Junbao Li

**Affiliations:** Information Countermeasure Technique Institute, Faculty of Computing, Harbin Institute of Technology, Harbin 150000, China; 21b905013@stu.hit.edu.cn (F.X.); 25s103555@stu.hit.edu.cn (J.W.); 25s103551@stu.hit.edu.cn (B.P.); lijunbao@hit.edu.cn (J.L.)

**Keywords:** intelligent radar, multi-agent reinforcement learning, semi-physical validation

## Abstract

**Highlights:**

**What are the main findings?**

- An AlphaZero-based dual-agent game framework is proposed to support the evolutionary learning of radar anti-jamming strategies through intelligent radar–jammer interactions.
- Monte Carlo Tree Search is incorporated to guide high-value interaction sampling and improve the learning efficiency of the radar agent.

**What are the implications of the main findings?**

- The proposed method improves the sampling efficiency of high-value samples during interactions, accelerates the convergence of the radar agent in the training stage.
- The semi-physical validation bridges the gap between algorithmic simulation and practical radar–jamming system implementation.

**Abstract:**

Adaptive waveform decision-making remains a key challenge in radar anti-jamming, especially when radar systems need to evolve their strategies through dynamic confrontation with intelligent jammers. This paper presents an intelligent jammer-assisted radar anti-jamming evolution method based on the AlphaZero framework. The radar and jammer are modeled as two competing agents, and their waveform-level interaction is formulated as a sequential decision-making game. By combining self-play learning with Monte Carlo Tree Search, the proposed framework guides the generation of high-value interaction samples and improves the training efficiency of the radar agent. As a result, the radar agent can progressively optimize its anti-jamming strategy and enhance its decision-making capability during adversarial interactions. To further evaluate its practical feasibility, a semi-physical hardware-in-the-loop validation platform is developed. Experimental results show that the proposed method accelerates the convergence of the radar agent, improves the utilization efficiency of valuable interaction samples, and bridges the gap between algorithmic simulation and practical radar–jamming system implementation.

## 1. Introduction

Modern radar systems are increasingly required to operate in complex and contested electromagnetic environments, where intelligent jammers can dynamically sense, learn, and respond to radar transmissions. In such environments, fixed or rule-based waveform scheduling strategies are often insufficient to maintain robust sensing performance. Adaptive waveform decision-making has therefore become a critical capability for cognitive radar systems, especially when facing jammers with adaptive behaviors.

The confrontation between radar and jammer can be naturally regarded as a dynamic sequential decision-making problem. The radar attempts to select suitable transmit waveforms to improve target detection, tracking, or parameter estimation performance, whereas the jammer aims to degrade radar performance by allocating jamming resources according to the observed radar behavior. This mutual adaptation forms a typical adversarial interaction process. Compared with conventional anti-jamming methods, game-based and reinforcement-learning-based approaches provide a promising framework for modeling the sequential decision-making relationship between the radar and jammer [1]. Through continuous interaction, both agents can update their strategies and improve their decision-making capabilities.

However, practical radar–jammer confrontation remains challenging for several reasons. First, waveform-level decision-making usually involves a large action space, since the radar waveform may be characterized by multiple parameters such as carrier frequency, pulse width, bandwidth, pulse repetition interval, and modulation type. The jammer may also select different jamming patterns, power allocation strategies, and waveform-matching schemes. Second, the interaction between radar and jammer is sequential and highly coupled. The decision of one side directly affects the state transition, reward feedback, and subsequent strategy update of the other side, which makes independent optimization insufficient. Third, reinforcement learning (RL) methods often require a large number of interaction samples to obtain stable policies [2]. In radar–jammer confrontation, low-quality or redundant interaction samples may reduce learning efficiency, slow down early-stage convergence, and increase the training cost of the radar agent. Therefore, improving the quality and efficiency of sample generation is essential for intelligent radar–jammer game decision-making.

Existing studies on radar anti-jamming decision-making have explored various methods, including game theory, Markov decision processes, RL, and multi-agent reinforcement learning (MARL) [3]. These methods have demonstrated their effectiveness in improving radar adaptability under different jamming scenarios. Nevertheless, many existing studies mainly focus on simulation environments, where the radar–jammer interaction process, signal transmission, and jamming effects are often simplified. Although simulation-based evaluation is useful for algorithm design and preliminary validation, it cannot fully reflect the constraints and uncertainties of practical systems, such as real-time waveform generation, signal processing delay, hardware interface limitations, and closed-loop interaction requirements. Therefore, there remains a gap between algorithmic simulation and practical implementation for intelligent radar–jammer game decision-making.

To address these issues, this paper proposes an AlphaZero-based dual-agent RL method for waveform-level radar–jammer interaction. AlphaZero combines self-play learning with search-based decision-making, making it suitable for adversarial sequential decision problems. In the proposed framework, the radar and jammer are modeled as two competing agents, and their interaction is formulated as a sequential game process. Through self-play, the two agents iteratively improve their policies during mutual confrontation. Meanwhile, Monte Carlo Tree Search is employed to guide action selection and enhance decision evaluation. By focusing the search process on high-value actions and trajectories, Monte Carlo Tree Search improves the quality and efficiency of generated interaction samples, accelerates early-stage policy convergence, and reduces the training cost of the radar agent.

Different from conventional single-agent waveform optimization methods, the proposed method emphasizes mutual learning and competitive evolution between radar and jammer. The radar does not merely optimize its waveform against a fixed jamming model; instead, it learns to make waveform decisions while the jammer simultaneously updates its own jamming strategy. This dual-agent formulation better reflects the interactive nature of intelligent radar–jammer confrontation. Moreover, the proposed method is designed at the waveform decision level, which provides a flexible framework for incorporating different waveform and jamming action sets.

**Figure 1:**
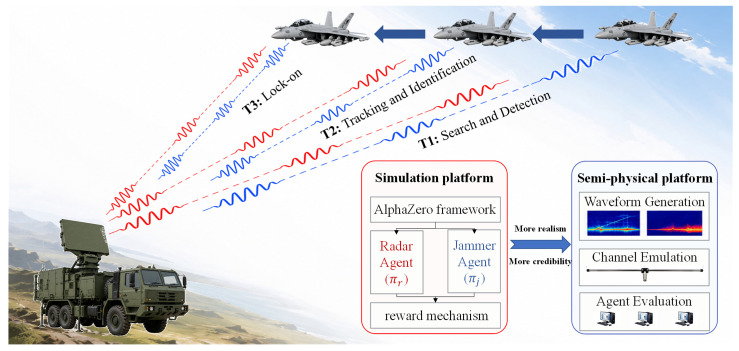
Research background of radar anti-jamming procedure.

In addition to the algorithmic framework, this paper develops a semi-physical platform to evaluate the feasibility of the proposed method in a more realistic experimental environment. The platform integrates algorithmic decision-making, waveform generation, signal transmission, jamming control, and performance evaluation into a closed-loop radar–jammer interaction process. Compared with pure numerical simulation, the semi-physical platform provides a more practical validation route by introducing hardware constraints and real signal interaction characteristics. Waveform-level radar–jammer game experiments are carried out on the platform to verify the effectiveness of the proposed framework. The research background is shown in Figure 1.

The main contributions of this paper are summarized as follows.

- An AlphaZero-based dual-agent RL framework is proposed for waveform-level radar–jammer confrontation. The radar and jammer are modeled as competing agents, and their interaction is formulated as a sequential game process.
- A self-play learning and Monte Carlo Tree Search-based decision-making mechanism is developed to regulate the mutual learning process between the radar and jammer. The proposed method improves the quality and efficiency of interaction sample generation, accelerates early-stage policy convergence, and reduces the training cost of the radar agent.
- A semi-physical platform is constructed for radar–jammer game validation. The platform provides a feasible experimental route from algorithmic simulation to semi-physical implementation. The results demonstrate that the method can achieve effective mutual game control between radar and jammer with limited interaction data and improve the feasibility of intelligent decision-making in realistic electromagnetic environments.

## 2. Related Work

Adaptive radar anti-jamming has been widely studied from the perspectives of waveform optimization, intelligent decision-making, game-theoretic modeling, and experimental validation. This section reviews representative studies related to the proposed work and discusses their limitations in waveform-level radar–jammer confrontation, particularly in terms of interaction efficiency, sample quality, and practical validation.

### 2.1. Adaptive Waveform Design for Radar Anti-Jamming

Waveform design is a fundamental technique for improving radar sensing performance in contested electromagnetic environments. Traditional anti-jamming waveform methods mainly focus on frequency agility, pulse parameter adjustment, waveform diversity, and optimization-based waveform design [4]. By adjusting waveform parameters such as carrier frequency, bandwidth, pulse width, pulse repetition interval, and modulation mode, these methods can improve radar robustness against specific jamming patterns. For example, game-theoretic waveform design methods have been used to model the conflict between radar and jammer, where the radar optimizes its transmit waveform according to the possible response of the jammer [5]. These studies demonstrate that adaptive waveform selection can effectively improve radar performance compared with fixed waveform strategies.

However, most conventional waveform optimization methods rely on prior knowledge of the jammer model or a predefined objective function [6]. When the jammer is intelligent and can dynamically adjust its strategy according to radar behavior, the optimal waveform decision may vary over time. In addition, waveform-level decision-making usually involves a large action space, since both radar waveforms and jamming strategies can be characterized by multiple parameters [7]. Static optimization methods are therefore insufficient for capturing the continuous and competitive evolution between radar and jammer [8]. Learning-based adaptive decision-making has consequently become an important direction for radar anti-jamming research.

### 2.2. Learning Based Radar–Jammer Interaction

Reinforcement learning has been widely applied to radar anti-jamming decision-making because it enables adaptive strategies to be learned through interaction with the environment. Early studies formulated radar anti-jamming as a Markov decision process and applied Q-learning or deep Q-network methods to frequency-hopping and waveform-switching problems [9,10]. With the increasing complexity of electromagnetic environments, more advanced deep reinforcement learning methods, including actor–critic, policy-gradient, and deep decision-making networks, were subsequently introduced for waveform design, jamming suppression, and resource allocation [2,11,12].

However, many existing reinforcement-learning-based studies still treat the jammer as part of the environment or assume a fixed jamming strategy. Such an assumption is insufficient for intelligent radar–jammer confrontation, in which both sides may continuously observe, learn, and adapt their policies during interaction [13,14]. To better characterize this competitive relationship, game-theoretic methods and multi-agent reinforcement learning have been introduced into radar–jammer decision-making [5,14,15]. More broadly, reinforcement learning for non-cooperative games has established an important theoretical foundation for competitive multi-agent decision-making. Representative methods include Minimax-Q for zero-sum stochastic games [16], Nash-Q for general-sum games [17], and Friend-or-Foe Q-learning [18], which simplifies opponent modeling by distinguishing cooperative and adversarial agents. By incorporating game-theoretic solution concepts into value updates, these methods provide important insights into adaptive competition among multiple decision-makers.

Nevertheless, most classical non-cooperative reinforcement-learning methods are developed for finite state and action spaces and often require equilibrium computation over discrete joint actions. Directly extending them to waveform-level radar–jammer confrontation is therefore challenging, because practical radar and jamming decisions usually involve multiple coupled parameters and hybrid discrete–continuous action spaces. In addition, radar–jammer interaction is inherently sequential: a radar waveform decision changes the jammer’s observation and response, while the resulting jamming action further affects the subsequent radar observation and decision [13,14]. Reinforcement learning for sequential decision-making therefore provides an important methodological basis for modeling this explicit action–response relationship.

Despite these advances, waveform-level radar–jammer games still face two major challenges. First, neural-network-based policy learning generally requires a large number of interaction samples to obtain stable strategies, while low-quality or redundant samples can reduce learning efficiency and slow early-stage convergence [2,19]. Second, purely policy-driven methods usually lack explicit look-ahead evaluation of future interaction consequences, which limits their ability to identify high-value waveform and jamming actions in strongly coupled sequential confrontation.

Monte Carlo Tree Search provides a natural mechanism for addressing these limitations. By selectively expanding promising action trajectories and evaluating potential future outcomes, MCTS can guide agents toward more valuable interactions rather than relying solely on random exploration or direct policy output [20]. When combined with self-play learning, MCTS can further improve sample quality, enhance action evaluation, and accelerate policy optimization. AlphaZero integrates self-play reinforcement learning with MCTS and provides an effective framework for combining policy learning with look-ahead search in competitive sequential decision-making.

Inspired by this mechanism, the present study develops a dual-agent framework specifically for waveform-level radar–jammer confrontation. Unlike classical non-cooperative reinforcement-learning methods that primarily rely on equilibrium-based value updates, the proposed method uses MCTS to selectively evaluate future radar–jammer interaction trajectories and to generate search-enhanced policy targets during self-play. The resulting framework is designed to improve the utilization of high-value interaction samples in a large waveform decision space and is further validated through a semi-physical closed-loop platform.

### 2.3. Simulation and Semi-Physical Validation

Most existing learning-based radar anti-jamming methods are validated in numerical simulation environments. Simulation experiments are useful for algorithm design and performance comparison, but they often simplify signal generation, jamming effects, hardware delay, synchronization error, hardware interface constraints, and closed-loop interaction requirements. Therefore, performance obtained in simulation may not fully reflect the feasibility of practical radar systems.

Semi-physical hardware-in-the-loop validation provides a more realistic experimental route between software simulation and field testing. Recent studies on radar jamming simulators and software-defined radar/jammer systems have shown that hardware-involved platforms can reproduce more practical signal transmission and jamming conditions [21]. By integrating waveform generation, signal transmission, jamming control, signal processing, and decision-making algorithms into a closed loop, semi-physical platforms can evaluate not only algorithmic performance but also implementation feasibility [22].

However, existing semi-physical validation studies mainly focus on signal-level verification, jamming effect reproduction, or predefined anti-jamming strategies. There are still relatively few studies that combine intelligent radar–jammer game decision-making with semi-physical hardware-in-the-loop validation. In particular, the validation of AlphaZero-based mutual game learning and Monte Carlo Tree Search-guided waveform decision-making under hardware-involved interaction remains insufficient. Therefore, this paper develops a semi-physical validation platform to support waveform-level radar–jammer game experiments and to provide a feasible route from algorithmic simulation to semi-physical implementation.

## 3. AlphaZero-Based Dual-Agent Reinforcement Learning Method

### 3.1. Problem Formulation of Radar–Jammer Confrontation

In this paper, the waveform-level confrontation between an intelligent radar and an intelligent jammer is formulated as a two-agent sequential game because both sides possess independent sensing and decision-making capabilities, while their objectives are directly opposed. The radar seeks to maintain target-detection performance by adapting its waveform type and waveform parameters, whereas the jammer attempts to degrade radar performance by adjusting its jamming type and jamming parameters. Since the jammer can change its strategy according to the intercepted radar waveform, it cannot be regarded as a fixed environmental disturbance. Similarly, the radar continuously updates its anti-jamming behavior according to the observed jamming effect. Therefore, modeling both sides as adaptive agents provides a more realistic representation of intelligent radar–jammer confrontation.

Although the radar and jammer operate according to their own local time perspectives at the waveform level, their behavioral changes are strongly coupled. In practice, a change in the radar waveform usually triggers a corresponding adjustment in the jammer strategy, while a change in the jamming behavior further induces the radar to modify its subsequent waveform. Therefore, the interaction is modeled as a sequential decision-making process rather than as two isolated or completely simultaneous decision processes. In each interaction cycle, the radar waveform decision influences the jammer observation and response, and the resulting jamming action further affects the radar echo, detection performance, and next waveform decision. This sequential formulation makes it possible to explicitly describe the causal relationship between jamming and anti-jamming behaviors, and is more suitable for exploring their underlying behavioral logic and strategy evolution over multiple interaction stages.

Let the radar agent and jammer agent be denoted by R and J, respectively. At the t-th decision step, the radar selects a waveform action $a_{t}^{R}\in A^{R}$, and the jammer selects a jamming action $a_{t}^{J}\in A^{J}$. The radar action consists of a waveform type and its corresponding parameters, which can be written as

$$a_{t}^{R}=\{m_{t}^{R},\eta_{t}^{R}\},$$

where $m_{t}^{R}\in \{LFM,CP\}$ denotes the radar waveform type. For the LFM action, the transmit waveform is generated as

$$x_{t,LFM}^{R}(\tau )=A_{R}rect\left(\frac{\tau }{T_{p}}\right)exp\left\{j2\pi \left[\left(f_{t}^{R}-\frac{B_{t}^{R}}{2}\right)\tau +\frac{B_{t}^{R}}{2T_{p}}\tau^{2}\right]\right\},$$

where $A_{R}$ is the waveform amplitude and $T_{p}$ is the fixed pulse width. The instantaneous frequency increases linearly from $f_{t}^{R}-B_{t}^{R}/2$ to $f_{t}^{R}+B_{t}^{R}/2$, so the Actor output $f_{t}^{R}$ determines the center frequency and $B_{t}^{R}$ determines the occupied bandwidth. The parameter vector is

$$\eta_{t,LFM}^{R}=\{f_{t}^{R},B_{t}^{R}\},$$

**Figure 2:**
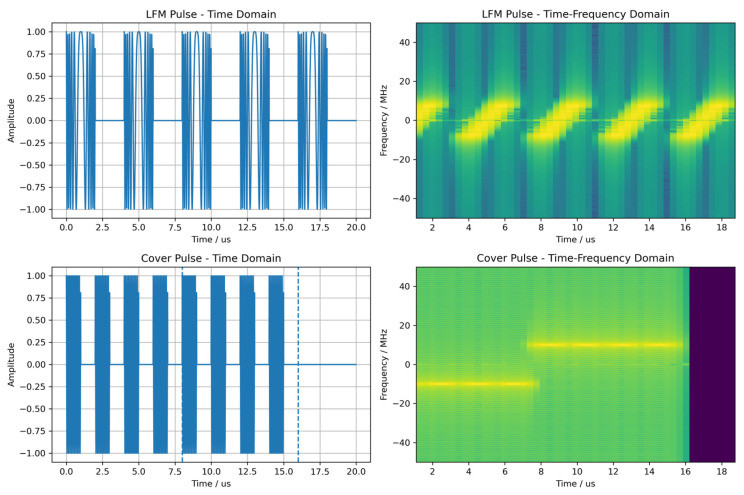
Time-Domain and Time-Frequency Plots of Radar Anti-Jamming Waveforms.

For the cover-pulse action, the transmitted waveform consists of a cover pulse followed by a detection pulse:

$$x_{t,CP}^{R}(\tau )=\left\{\begin{array}{ll} A_{c}exp\left(j2\pi f_{t,c}^{R}\tau \right), & 0\leq \tau <T_{t,c}^{R},\\ A_{d}exp\left[j2\pi f_{t,d}^{R}\left(\tau -T_{t,c}^{R}\right)\right], & T_{t,c}^{R}\leq \tau <T_{t,c}^{R}+T_{t,d}^{R},\\ 0, & \mathrm{otherwise}, \end{array}\right.$$

where the cover pulse is transmitted first to induce or attract the jammer response, while the subsequent detection pulse is used for target sensing. The four adjustable parameters therefore determine the frequencies and durations of the two pulse segments. The parameter vector is

$$\eta_{t,CP}^{R}=\{f_{t,c}^{R},f_{t,d}^{R},T_{t,c}^{R},T_{t,d}^{R}\},$$

where $f_{t,c}^{R}$ and $f_{t,d}^{R}$ denote the carrier frequencies of the cover pulse and the detection pulse, respectively. $T_{t,c}^{R}$ and $T_{t,d}^{R}$ denote the cover-pulse width and the detection-pulse width, respectively. The cover pulse is transmitted to mislead or mask the jammer, while the detection pulse is used for actual radar sensing. The simulation results of time and time frequency domains of radar anti-jamming waveforms are shown in Figure 2.

The jammer action also consists of a jamming type and its corresponding parameters, which can be written as

$$a_{t}^{J}=\{m_{t}^{J},\eta_{t}^{J}\},$$

where $m_{t}^{J}\in \{NBJ,RDJ\}$ denotes narrowband blocking jamming, and repeater deception jamming, respectively. For NBJ, the jammer generates band-limited complex noise centered at the selected jamming frequency:

$$j_{t,NBJ}^{J}(\tau )=\sqrt{P_{J}}u_{B_{t}^{J}}(\tau )exp\left(j2\pi f_{t}^{J}\tau \right),$$

where $u_{B_{t}^{J}}(\tau )$ denotes a zero-mean complex Gaussian noise sequence filtered to bandwidth $B_{t}^{J}$, and $P_{J}$ denotes the jamming power. Therefore, $f_{t}^{J}$ and $B_{t}^{J}$ control the center frequency and spectral coverage of the suppression jamming. The adjustable parameters are the jamming frequency and bandwidth:

$$\eta_{t,NBJ}^{J}=\{f_{t}^{J},B_{t}^{J}\}.$$

For RDJ, the jammer intercepts the radar waveform and retransmits multiple delayed copies:

$$j_{t,RDJ}^{J}(\tau )=\sum_{n=1}^{N_{t,rep}^{J}}\;\beta_{n}{\hat{x}}_{t}^{R}\left(\tau -T_{t,rec}^{J}-(n-1)\Delta T_{rep}\right),$$

where ${\hat{x}}_{t}^{R}$ is the intercepted radar signal, $\beta_{n}$ is the amplitude coefficient of the n-th forwarded copy, $\Delta T_{\mathrm{rep}}$ is the forwarding-delay interval and $T_{t,rec}^{J}$ denotes the time used by the jammer to intercept the radar signal. Increasing $N_{t,\mathrm{rep}}^{J}$ generates more delayed replicas and therefore more false-target echoes. The adjustable parameters are the interception time and the number of forwarding repetitions:

$$\eta_{t,RDJ}^{J}=\{T_{t,rec}^{J},N_{t,rep}^{J}\},$$

**Figure 3:**
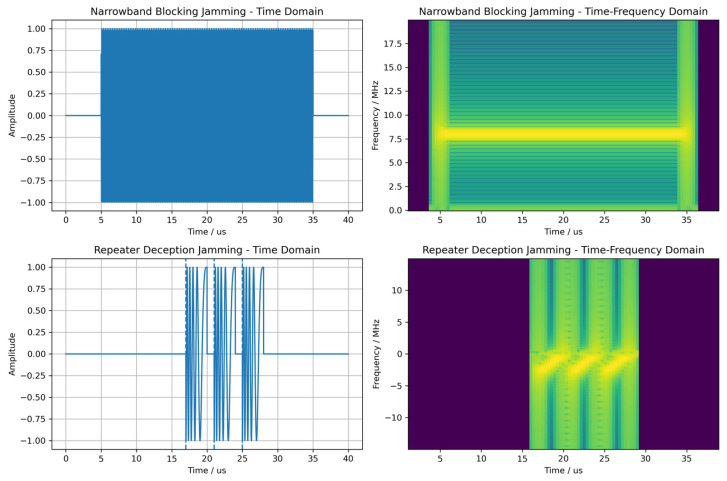
Time-Domain and Time-Frequency Plots of Jammer Jamming Waveforms.

At each decision step, the radar first selects a waveform action and generates the transmit signal $x_{t}^{R}\left(\tau \right)$. The jammer then intercepts the radar signal, selects a jamming action, and generates the jamming signal $j_{t}^{J}\left(\tau \right)$. The mixed signal received by the radar can be expressed as

$$y_{t}\left(\tau \right)=\alpha_{t}x_{t}^{R}\left(\tau -\tau_{t}\right)exp\left(j2\pi f_{d,t}\tau \right)+j_{t}^{J}\left(\tau \right)+n_{t}\left(\tau \right),$$

where $\alpha_{t}$, $\tau_{t}$, and $f_{d,t}$ denote the target echo amplitude, delay, and Doppler frequency, respectively, and $n_{t}\left(\tau \right)$ denotes noise. The simulation results of time and time frequency domains of jamming waveforms are shown in Figure 3.

A competitive reward is used to describe the opposite objectives of the radar and the jammer. Since this paper focuses on radar detection performance under jamming, the normalized detection probability is used as the main reward criterion throughout the experiments. The radar reward is defined as

$$r_{t}^{R}={\bar{P}}_{d,t},$$

where ${\bar{P}}_{d,t}$ denotes the normalized detection probability of the radar at the t-th decision step. The jammer aims to degrade the radar detection performance; therefore, its reward is defined as the opposite of the radar reward:

$$r_{t}^{J}=-r_{t}^{R}.$$

This simplified detection-based reward allows the radar–jammer game to be evaluated directly from the perspective of radar sensing performance.

### 3.2. AlphaZero-Based Dual-Agent Reinforcement Learning Framework

**Figure 4:**
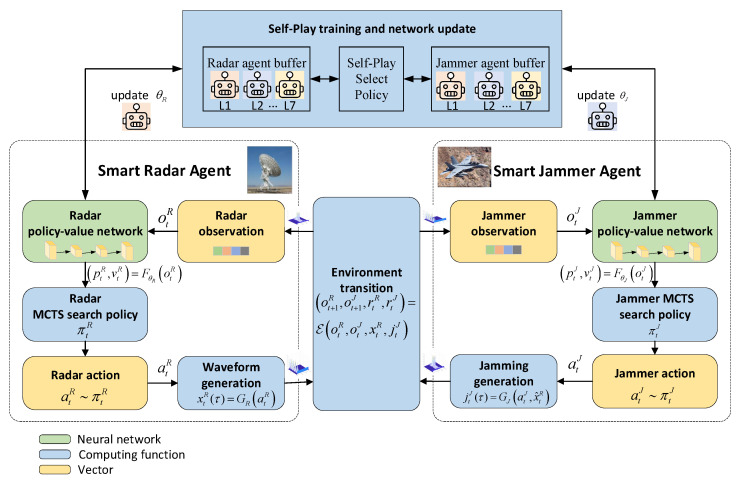
The framework of AlphaZero-based dual-agent RL method.

To solve the waveform-level radar–jammer confrontation problem, an AlphaZero-based dual-agent game framework is proposed (Figure 4). The framework combines policy-value estimation, Monte Carlo tree search (MCTS), and self-play learning. In this framework, both the radar and the jammer are modeled as learning agents.

At each decision step, the closed-loop interaction includes five modules: radar decision-making, radar waveform generation, jammer interception and decision-making, jamming signal generation, and radar performance evaluation. First, the radar obtains its observation $o_{t}^{R}$ from the receiver and historical waveform information. The radar policy-value network outputs

$$\left(p_{t}^{R},v_{t}^{R}\right)=F_{\theta_{R}}\left(o_{t}^{R}\right),$$

where $p_{t}^{R}$ is the prior distribution over the radar action space and $v_{t}^{R}$ is the estimated value. Based on the network output, MCTS generates an improved radar policy $\pi_{t}^{R}$, and the radar action is selected as

$$a_{t}^{R}∼\pi_{t}^{R}.$$

The selected action is then converted into a radar transmit waveform by the waveform generation module:

$$x_{t}^{R}\left(\tau \right)=G_{R}\left(a_{t}^{R}\right),$$

where $G_{R}\left(\cdot \right)$ denotes the radar waveform generation function.

After radar transmission, the jammer intercepts part of the radar signal and forms its observation:

$$o_{t}^{J}=\Omega^{J}\left(x_{t}^{R}\left(\tau \right),h_{t-1}^{J},\xi_{t}^{J}\right),$$

where $h_{t-1}^{J}$ denotes historical jamming information and $\xi_{t}^{J}$ represents interception noise and delay. The jammer policy-value network outputs

$$\left(p_{t}^{J},v_{t}^{J}\right)=F_{\theta_{J}}\left(o_{t}^{J}\right).$$

MCTS is then used to obtain the improved jammer policy $\pi_{t}^{J}$, and the jammer action is selected as

$$a_{t}^{J}∼\pi_{t}^{J}.$$

According to the selected jamming type and parameters, the jamming generation module produces

$$j_{t}^{J}\left(\tau \right)=G_{J}\left(a_{t}^{J},{\hat{x}}_{t}^{R}\right),$$

where $G_{J}\left(\cdot \right)$ is the jamming signal generation function and ${\hat{x}}_{t}^{R}$ is the intercepted radar signal. Finally, the radar receiver obtains the mixed echo, evaluates the sensing performance, and returns the next observations and rewards:

$$\left(o_{t+1}^{R},o_{t+1}^{J},r_{t}^{R},r_{t}^{J}\right)=E\left(o_{t}^{R},o_{t}^{J},x_{t}^{R},j_{t}^{J}\right),$$

where $E\left(\cdot \right)$ denotes the closed-loop radar–jammer interaction environment.

Through this process, the radar decision affects waveform generation, the waveform affects jammer observation and jamming decision, and the jamming signal further affects radar reception and performance evaluation. The evaluated rewards are then used for self-play training. Therefore, the proposed framework enables both agents to improve their strategies through continuous radar–jammer confrontation.

### 3.3. Self-Play Training Process

The proposed method uses self-play to train the radar and jammer policies. In self-play, the radar and jammer repeatedly compete with each other using their current policies. The interaction data generated during this process are used to update the neural networks of both agents. As training proceeds, the radar learns to avoid or suppress effective jamming actions, while the jammer learns to exploit weaknesses in the radar waveform strategy.

For agent i∈{R,J}, the neural network is denoted as

$$F_{\theta_{i}}\left(o_{t}^{i}\right)=\left(p_{t}^{i},v_{t}^{i}\right),$$

where $p_{t}^{i}$ is the predicted policy, $v_{t}^{i}$ is the predicted value, and $\theta_{i}$ denotes the network parameters. The network $\theta_{i}$ is an improved DDPG-based network proposed in our previous work [23]. During self-play, Monte Carlo tree search is performed based on the current network, producing an improved policy $\pi_{t}^{i}$. At each decision step, the training sample is recorded as

$$d_{t}^{i}=\left(o_{t}^{i},\pi_{t}^{i},r_{t}^{i}\right).$$

After one episode terminates, the cumulative return of agent i from step t is calculated as

$$z_{t}^{i}=\sum_{k=t}^{T}\gamma^{k-t}r_{k}^{i},$$

where T is the length of the episode, and $\gamma \in \left[0,1\right]$ is the discount factor. The final training sample is then written as

$${\tilde{d}}_{t}^{i}=\left(o_{t}^{i},\pi_{t}^{i},z_{t}^{i}\right).$$

All samples generated by self-play are stored in the replay buffer:

$$D_{i}=\{\left(o_{t}^{i},\pi_{t}^{i},z_{t}^{i}\right)\}.$$

The value head is trained using the cumulative self-play return z as the supervised target. Therefore, the value-prediction loss is defined as

$$L_{v}^{i}={\left(z-v_{\theta_{i}}(o)\right)}^{2}.$$

The policy head is trained to reproduce the MCTS-enhanced policy π. Its loss is the cross-entropy between π and the network output $p_{\theta_{i}}$:

$$L_{p}^{i}=-\sum_{a}\;\pi (a)log\;p_{\theta_{i}}(a|o).$$

This term can also be written as

$$L_{p}^{i}=D_{KL}\left(\pi \|p_{\theta_{i}}\right)+H(\pi ),$$

where H(π) is independent of the network parameters. Therefore, minimizing the cross-entropy is equivalent to minimizing the Kullback–Leibler divergence between the MCTS policy and the network policy. In this way, the search result is progressively transferred to the policy network.

Adding $L_{2}$ regularization gives the mini-batch objective:

$$L^{i}\left(\theta_{i}\right)=\frac{1}{\left|B_{i}\right|}\sum_{(o,\pi ,z)\in B_{i}}\;\left[{\left(z-v_{\theta_{i}}(o)\right)}^{2}-\pi^{T}log\;p_{\theta_{i}}(o)+\lambda {\left\|\theta_{i}\right\|}_{2}^{2}\right].$$

The first term trains long-term outcome prediction, the second distills the MCTS action preference into the policy network, and the third limits excessive parameter growth.

The parameters are updated by gradient descent:

$$\theta_{i}\leftarrow \theta_{i}-\eta \nabla_{\theta_{i}}L_{i}\left(\theta_{i}\right),$$

where η is the learning rate. Since the radar and jammer have opposite objectives, their rewards are competitive. In the zero-sum setting, the relationship is

$$z_{t}^{J}=-z_{t}^{R}.$$

Therefore, the optimization process makes the radar policy evolve toward better anti-jamming performance, while the jammer policy evolves toward stronger interference capability. The self-play process can be expressed as the following iterative optimization problem:

$$\theta_{R}^{k+1}=arg\underset{\theta_{R}}{min}L_{R}\left(\theta_{R};\theta_{J}^{k}\right),$$

$$\theta_{J}^{k+1}=arg\underset{\theta_{J}}{min}L_{J}\left(\theta_{J};\theta_{R}^{k}\right),$$

where k denotes the training iteration. In each iteration, the radar policy is optimized against the current jammer policy, and the jammer policy is optimized against the current radar policy. This alternating competitive learning mechanism enables both agents to improve through mutual confrontation.

### 3.4. Monte Carlo Tree Search

**Figure 5:**
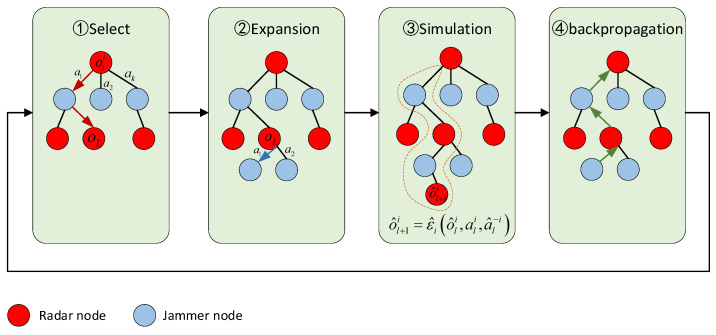
The computing step of Monte Carlo tree search.

Monte Carlo tree search is used to enhance the decision-making process of both radar and jammer. Compared with directly selecting actions from the neural network policy, MCTS evaluates candidate actions by considering possible future interactions. The computing step of Monte Carlo tree search is shown in Figure 5.

For agent i∈{R,J}, MCTS is performed from the current observation node $o_{t}^{i}$. Each edge $\left(o,a\right)$ in the search tree stores four quantities: the prior probability $P\left(o,a\right)$, visit count $N\left(o,a\right)$, accumulated value $W\left(o,a\right)$, and mean action value $Q\left(o,a\right)$. The mean action value is defined as

$$Q\left(o,a\right)=\frac{W\left(o,a\right)}{N\left(o,a\right)}.$$

At the beginning of the search, the neural network provides the prior probability and value estimate:

$$\left(P\left(o,\cdot \right),v\left(o\right)\right)=F_{\theta_{i}}\left(o\right).$$

The action-selection criterion is constructed by combining an exploitation term and a prior-guided exploration term:

$$a^{*}=arg\;\underset{a}{max}\;[Q(o,a)+U(o,a)],$$

where

$$U(o,a)=c_{puct}P(o,a)\frac{\sqrt{N(o)}}{1+N(o,a)},N(o)=\sum_{b}\;N(o,b).$$

The mean value Q(o,a) favors actions that have achieved high predicted returns in previous simulations. The exploration bonus U(o,a) is large when the policy prior P(o,a) is high and the edge has been visited only a few times. As N(o,a) increases, the exploration bonus decreases, and the selection becomes increasingly dominated by the accumulated value estimate. Therefore, the PUCT rule first uses the policy network to focus the search on promising waveform actions and then refines their ranking through repeated radar–jammer interaction evaluation.

The proposed action is hybrid, consisting of a discrete waveform or jamming type and the corresponding continuous parameters. The policy head first outputs the probability of each action type using a softmax function. The continuous parameter head outputs normalized values through a hyperbolic tangent function. Each normalized output ${\tilde{\eta }}_{t,j}^{i}\in [-1,1]$ is mapped to its physical range according to

$$\eta_{t,j}^{i}=\eta_{j,min}^{i}+\frac{{\tilde{\eta }}_{t,j}^{i}+1}{2}\left(\eta_{j,max}^{i}-\eta_{j,min}^{i}\right).$$

To obtain a finite branching set for MCTS, the continuous outputs are quantized according to the hardware-control resolution, and only the K candidate actions with the highest policy priors are retained at each node. MCTS is therefore performed over a finite candidate set rather than over the entire continuous action domain.

After an action is selected, the search process uses the radar–jammer interaction model to generate the next observation node. For the radar search tree, a radar action is selected first, and the possible jammer response is estimated using the current jammer policy. For the jammer search tree, a jammer action is selected based on its current observation, and the possible radar response in subsequent steps is estimated using the current radar policy. This opponent-policy-guided simulation can be written as

$${\hat{o}}_{l+1}^{i}={\hat{E}}_{i}\left({\hat{o}}_{l}^{i},a_{l}^{i},{\hat{a}}_{l}^{-i}\right),$$

where ${\hat{E}}_{i}\left(\cdot \right)$ denotes the internal simulation model used by agent i, $a_{l}^{i}$ is the action selected by the searching agent, and ${\hat{a}}_{l}^{-i}$ is the predicted opponent action generated by the opponent policy network. This allows MCTS to estimate the influence of future radar–jammer interactions.

When a leaf node is reached, it is expanded using the neural network:

$$\left(P\left(o_{L},\cdot \right),v_{L}\right)=F_{\theta_{i}}\left(o_{L}\right),$$

where $o_{L}$ is the leaf observation node and $v_{L}$ is the estimated value. The value is then backed up along the selected path. For each visited edge $\left(o_{l},a_{l}\right)$, the statistics are updated as

$$N\left(o_{l},a_{l}\right)\leftarrow N\left(o_{l},a_{l}\right)+1,$$

$$W\left(o_{l},a_{l}\right)\leftarrow W\left(o_{l},a_{l}\right)+v_{L},$$

$$Q\left(o_{l},a_{l}\right)\leftarrow \frac{W\left(o_{l},a_{l}\right)}{N\left(o_{l},a_{l}\right)}.$$

After M simulations, the improved policy is obtained from the visit counts:

$$\pi_{t}^{i}\left(a\right)=\frac{N{\left(o_{t}^{i},a\right)}^{1/\tau }}{\sum_{b}N{\left(o_{t}^{i},b\right)}^{1/\tau }},$$

where τ is the temperature parameter. A larger τ encourages exploration, while a smaller τ makes the policy closer to greedy action selection. During training, actions are sampled from $\pi_{t}^{i}$ to maintain exploration. During testing or semi-physical implementation, the action with the largest visit count can be selected:

$$a_{t}^{i}=arg\underset{a}{max}N\left(o_{t}^{i},a\right).$$

In the proposed radar–jammer game, MCTS plays two roles. First, it transforms the raw network policy into a search-enhanced policy, improving the quality of waveform and jamming decisions. Second, it provides a better training target for the policy network. By learning from the MCTS-generated policy, the neural network gradually internalizes the search result and improves its decision-making capability.

The complete procedure of the proposed method is summarized in Algorithm 1.
**Algorithm 1** AlphaZero-based dual-agent radar-jammer game control method**Require:** Radar action space
$A^{R}$, jammer action space
$A^{J}$, initial network parameters
$\theta_{R}$ and
$\theta_{J}$, number of training episodes
N, number of MCTS simulations
M.
**Ensure:** Trained radar policy network
$F_{\theta_{R}}$ and jammer policy network
$F_{\theta_{J}}$.
1:Initialize the radar network $F_{\theta_{R}},$ jammer network $F_{\theta_{J}},$ and replay buffers.2:**for** *episode* = 1 to *N* **do**3:    Initialize the radar-jammer confrontation environment.4:    **for** each decision step t **do**5:          Obtain incomplete observations $o_{t}^{R}$ and $o_{t}^{J}.$
6:          Use $F_{\theta_{R}}$ and MCTS with M simulations to obtain radar search policy $\pi_{t}^{R}.$
7:          Use $F_{\theta_{J}}$ and MCTS with M simulations to obtain jammer search policy $\pi_{t}^{J}.$
8:          Select radar waveform action $a_{t}^{R}$ according to $\pi_{t}^{R}.$
9:          Select jammer action $a_{t}^{J}$ according to $\pi_{t}^{J}.$
10:          Execute $a_{t}^{R}$ and $a_{t}^{J}$ in the radar-jammer interaction environment.11:          Calculate rewards $r_{t}^{R}$ and $r_{t}^{J},$ and update the environment state.12:          Store observations and search policies.13:    **end for**
14:    Calculate final returns $z^{R}$ and $z^{J}$ for the radar and jammer.15:    Store training samples $\left(o_{t}^{R},\pi_{t}^{R},z_{t}^{R}\right)$ and $\left(o_{t}^{J},\pi_{t}^{J},z_{t}^{J}\right)$ in replay buffers.16:    Update $F_{\theta_{R}}$ and $F_{\theta_{J}}$ by minimizing their loss functions.17:**end for**18:**return** The trained radar and jammer networks.

## 4. Implementation of the Semi-Physical Platform

A semi-physical validation platform for intelligent radar–jammer games is developed in this work. Based on a modular hardware architecture, the platform integrates intelligent decision-making algorithms, waveform generation modules, signal transmission and reception modules, and target/jamming environment simulation modules into a unified closed-loop validation system. This enables dynamic game interactions between the radar and jammer over a realistic signal transmission chain. This chapter first presents the overall architecture of the platform, followed by an analysis of its hardware configuration and closed-loop interaction mechanism.

### 4.1. Architecture Design of the Semi-Physical Validation Platform

**Figure 6:**
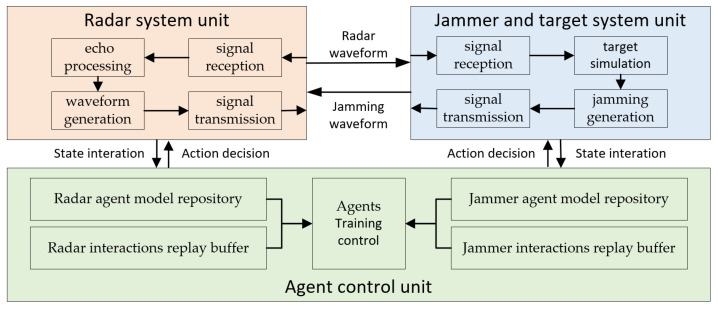
Semi-Physical Verification Platform Architecture.

The overall architecture of the platform consists of three parts: the radar system unit, the target and jamming system unit, and the agent control unit. The architecture design is shown in Figure 6.

The radar system unit is mainly responsible for radar waveform generation, signal transmission, signal reception, and echo processing. According to the control commands generated by the intelligent decision-making algorithm, the system generates the corresponding waveform in real time and processes the received target echoes and jamming signals.

The target and jamming system unit is used to construct the radar confrontation environment. The target simulation module generates target echo signals, while the jamming simulation module generates different types of jamming signals, such as suppression jamming and deception jamming, and dynamically adjusts the jamming parameters according to the decision results of the intelligent jammer agent.

The agent control unit serves as the control center of the platform and is responsible for running the intelligent decision-making algorithm, controlling the experimental procedure, and exchanging data among the functional modules. The agent control unit obtains radar state information and jamming state information in real time, calculates the current optimal strategy through the intelligent decision-making algorithm, and issues control commands to the radar and jamming systems.

During platform operation, the radar and jammer form a closed-loop interaction through the target and jamming environment. The radar selects the transmitted waveform according to the environmental state, while the jammer selects its jamming strategy according to the observed information. Both sides continuously adjust their strategies during confrontation, thereby enabling the real-time evolution of the intelligent game process.

**Figure 7:**
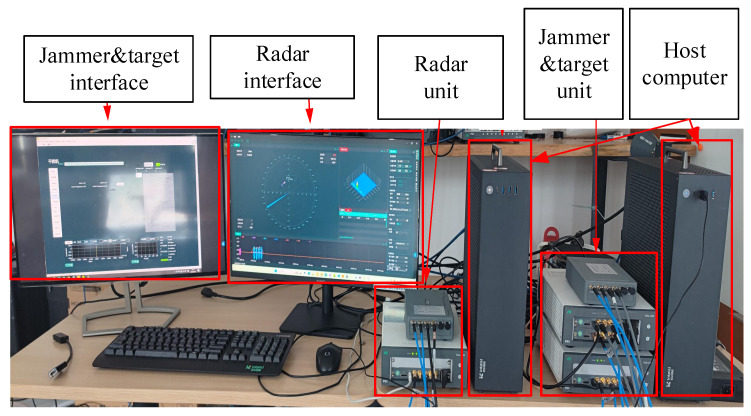
Semi-Physical Hardware Platform Composition.

The semi-physical hardware platform is constructed as shown in Figure 7. The proposed algorithm described in Algorithm 1 is directly deployed in the agent control unit of the semi-physical platform. As shown in Figure 7, the agent control unit runs on a Windows-based host computer and is connected to the radar, target, and jamming hardware units through wired communication interfaces. During each closed-loop interaction cycle, the host computer receives the processed radar and jamming state information, executes the policy-value networks and MCTS-based strategy computation, and generates the corresponding radar waveform and jamming actions. These actions are then converted into hardware-executable commands and transmitted to the relevant RF and waveform-generation modules. After signal transmission, acquisition, and processing, the resulting performance information is returned to the host computer for the next decision cycle. Therefore, Algorithm 1 constitutes the actual intelligent decision-making procedure used in the semi-physical hardware-in-the-loop experiments.

### 4.2. Hardware and Software Composition of the Platform

#### 4.2.1. Hardware Composition

**Figure 8:**
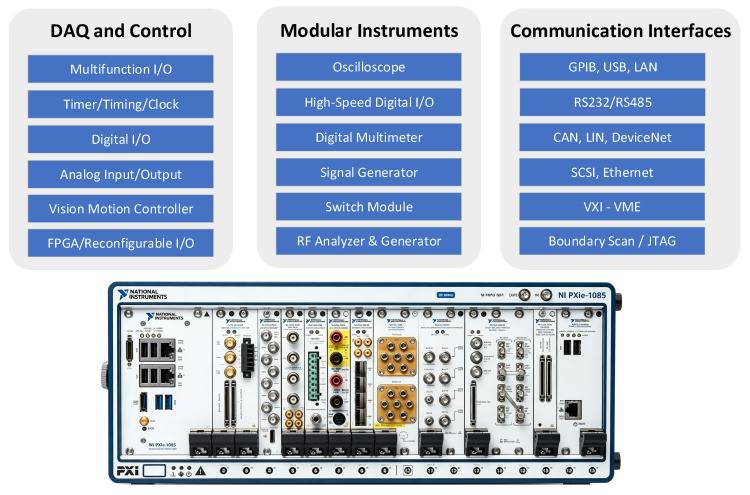
Modular Hardware Processing Platform Architecture.

The platform hardware mainly consists of a PXIe chassis, an embedded controller, radio-frequency (RF) transceiver modules, high-speed acquisition modules, arbitrary waveform generation modules, and FPGA/GPU processing modules. The PXIe chassis serves as the core carrier of the platform, providing a unified environment for power supply, thermal management, clock distribution, triggering, and bus interconnection for all functional modules. High-speed data exchange among the modules is implemented through the PXIe backplane bus, while a unified synchronization mechanism ensures coordinated operation among multiple modules. The modular hardware platform architecture is shown in Figure 8.

The RF transceiver modules are used to construct the transmission and reception links for radar and jamming signals. In the transmit link, the corresponding radar waveform or jamming waveform is generated according to the intelligent decision-making results and then output to the target and jamming environment after up-conversion and related processing. In the receive link, target echoes and jamming signals are received, down-converted, and acquired, providing raw data for subsequent signal processing. The high-speed acquisition modules are responsible for intermediate-frequency or baseband signal acquisition, whereas the arbitrary waveform generation modules are used to generate the experimental signals required for frequency agility, waveform switching, suppression jamming, and deception jamming.

The FPGA/GPU processing modules mainly perform real-time signal processing and intelligent algorithm computation. The FPGA is suitable for low-latency processing tasks, such as digital down-conversion, filtering, pulse compression, and parameter extraction, while the GPU is used for neural network inference, strategy computation, and large-scale data analysis. Through flexible combinations of hardware modules, the platform can support semi-physical validation under different radar systems, jamming patterns, and experimental scenarios.

#### 4.2.2. Software Composition

**Figure 9:**
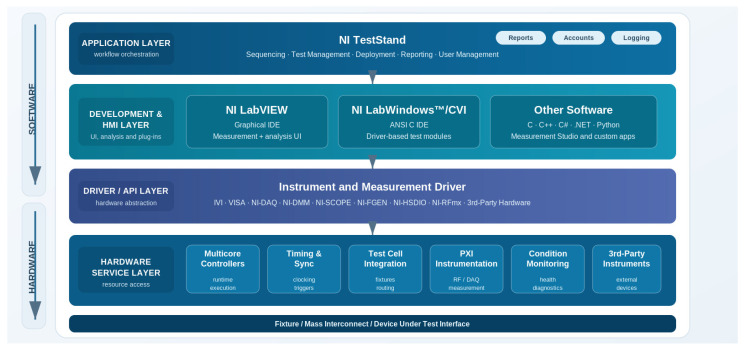
Modular Software Architecture.

The platform software adopts a layered modular design (Figure 9), mainly including the hardware driver layer, instrument control layer, signal processing layer, intelligent decision-making layer, and experiment management layer. The hardware driver layer is responsible for low-level access to various modules in the PXIe chassis and provides unified device interfaces for the upper-layer software. The instrument control layer is used to configure key parameters such as sampling rate, center frequency, bandwidth, transmit power, pulse repetition frequency, trigger mode, and synchronization clock, while monitoring the operating status of the hardware.

The signal processing layer processes the acquired target echoes and jamming signals, including down-conversion, filtering, target detection, jamming feature extraction, and environmental state construction. The processing results are used as observation information for the agents and are input to the intelligent decision-making layer. The intelligent decision-making layer runs the radar–jammer intelligent game algorithm, generates radar waveform selection results or jamming parameter configuration results according to the current state, and converts the decision results into hardware-executable control commands.

The experiment management layer is responsible for experimental procedure control, scenario configuration, round management, data recording, and result analysis. Through this software layer, the platform can uniformly manage radar transmission parameters, jamming parameters, received signals, strategy selection results, and performance metrics, thereby providing data support for subsequent algorithm evaluation and platform performance analysis.

### 4.3. Radar–Jammer Interaction Mechanism and Functional Design

To enable semi-physical validation of intelligent radar–jammer games, the platform establishes a closed-loop interaction chain composed of the radar module, jammer module, and target simulation module. The radar module generates transmission waveforms according to the decision results of the radar agent; the target simulation module modulates the radar signal to produce controllable target echoes; and the jammer module detects the radar signal and generates corresponding jamming signals based on the decisions of the jammer agent. These modules jointly implement a closed-loop process of radar transmission, target echo simulation, jamming signal generation, radar reception, and strategy updating.

#### 4.3.1. Radar Module

The radar module is responsible for waveform generation, RF transmission, echo reception, and signal processing. According to the hardware constraints, the controllable radar parameters include RF frequency, transmit attenuation, receive attenuation, and waveform type. The supported RF frequency range is 10 MHz–8 GHz, with one transmit channel and one receive channel. Within 550 MHz–8 GHz, the instantaneous bandwidth reaches 400 MHz, and the intermediate frequency is 750 MHz. The maximum RF input and output powers are both 0 dBm. The digital attenuation range is 90 dB for the transmit channel and 60 dB for the receive channel, both with a step size of 1 dB.

The radar module supports LFM signals and cover pulse signals. Based on the current environmental state and jamming observations, the radar agent can dynamically adjust the operating frequency, attenuation settings, and waveform type, thereby enabling frequency agility, waveform switching, and adaptive anti-jamming operation.

#### 4.3.2. Jammer Module

The jammer module is used to construct an intelligent jamming environment. It mainly includes a radar signal reconnaissance function, a suppression jamming signal generation function, and a deception jamming signal generation function.

The supported jamming types include narrowband blocking jamming and repeater deception jamming. For suppression jamming, the controllable parameters include jamming center frequency and signal bandwidth. For repeater deception jamming, the controllable parameters include sampling time, forwarding delay and forwarding times. The jammer module has the same RF link capability as the radar module, including a 10 MHz–8 GHz RF frequency range, one transmit and one receive channel, 400 MHz instantaneous bandwidth within 550 MHz–8 GHz, 750 MHz intermediate frequency, and 0 dBm maximum RF input and output powers.

#### 4.3.3. Target Simulation Module

The target simulation module is used to generate controllable target echoes and mainly consists of a digital radio frequency memory (DRFM) module, a target range simulation module, a target velocity simulation module, and a target radar cross-section (RCS) simulation module. The DRFM module stores and reconstructs the radar signal. The range simulation module applies a controllable time delay to emulate different target ranges. The velocity simulation module introduces Doppler frequency shifts through frequency modulation to simulate target motion. The RCS simulation module adjusts the echo amplitude to emulate different target scattering characteristics.

During closed-loop operation, the radar waveform is first transmitted to the target simulation module to generate the target echo, while the jammer generates jamming signals according to reconnaissance results. The radar receiver jointly processes the target echo and jamming signal, and the resulting state and performance feedback are returned to the radar and jammer agents for the next decision cycle. In this way, the platform realizes real-time closed-loop interaction among the radar, jammer, and target simulation modules.

## 5. Experiment

This chapter presents the experimental validation of the proposed method from two perspectives: simulation-based evaluation and semi-physical platform validation. Section 5.1 describes the parameter settings used in the experiments. Section 5.2 investigates whether the proposed method can learn an effective adaptive decision-making policy during the dynamic confrontation between the radar and the jammer. Section 5.3 compares the performance of the proposed algorithm with that of representative multi-agent reinforcement learning algorithms. Finally, Section 5.4 validates the practical feasibility of the proposed method on a semi-physical platform, where realistic signal generation, acquisition, processing, and closed-loop control are jointly considered.

### 5.1. Parameters Settings

In the simulation experiments, the waveform types and parameter ranges of the radar and the jammer are configured as listed in Table 1. The radar can schedule two types of waveforms, namely linear frequency-modulated (LFM) waveforms and cover signals. The jammer can schedule three types of jamming waveforms: narrowband blocking jamming and repeater deception jamming.

In the simulations, the radar cross section (RCS) is fixed at 3 m^2^. This setting is adopted because the RCS varies with the carrier frequency, and explicitly modeling this frequency-dependent variation would introduce a considerable computational burden. Moreover, the RCS has a limited influence on the evaluation metrics considered in this study. In contrast, the actual RCS of the physical target is considered in the semi-physical validation platform.

Part of the performance settings of the radar and the jammer used in the simulation experiments is summarized in Table 2. These parameters are selected with reference to the performance specifications of a practical radar system. It should be noted that these settings are not identical to those used in the hardware-in-the-loop validation platform. In addition, each agent model library contains seven models, indicating that the radar/jammer buffer can store agent models corresponding to seven different capability levels.

The detailed network architecture and the main training and search parameters are summarized in Table 3. Both the radar and jammer agents employ a policy-value network with a shared feature-extraction backbone and separate output heads. The policy component contains separate output heads for the discrete action type and the corresponding continuous parameters. In addition, the value head consists of a 240-neuron hidden layer and a scalar output used to estimate the long-term value of the current observation.

During training, the replay-buffer size is set to 10,000, the mini-batch size is 256, the learning rate is $1\times 10^{-4}$, and the discount factor is 0.99. Adam is used for network optimization. For MCTS, 100 simulations are performed for each decision process, the exploration coefficient $c_{\mathrm{puct}\;}$ is set to 1.5, and the temperature parameter is set to 1.0 during self-play training. During testing and semi-physical validation, the action with the largest MCTS visit count is selected to obtain a deterministic decision.

### 5.2. Adaptive Policy Learning in Dynamic Radar–Jammer Confrontation

In this experiment, the decision network parameters of the radar and the jammer are first initialized. During training, the detection probability is used as the main performance criterion for evaluating radar anti-jamming capability. The radar reward is directly calculated using the normalized detection probability, while the jammer reward is defined as its negative value. A successful radar interaction is counted when the detection probability exceeds the predefined detection threshold δ=0.6. The radar winning rate is then calculated as the ratio of successful interactions to the total number of evaluation rounds. When either agent achieves a winning rate higher than 55% over 100 consecutive confrontation rounds, the model parameters of that agent are fixed and saved. The saved model is then assigned a label denoted by L, which represents its capability level, such as an L1 radar or an L2 jammer.

For example, if the radar first reaches a winning rate of 55% in the initial game, the corresponding radar model is saved as the L1 radar. The parameters of this radar model are then fixed, and only the jammer network is trained to improve its confrontation capability until the jammer reaches the L1 capability level. Through this process of parameter freezing and alternating training, radar and jammer models from L1 to L7 are eventually obtained.

After obtaining the L1–L7 radar and jammer models, the confrontation capability is evaluated from the perspective of the radar. The evaluation metric is defined as the radar winning rate, whose value ranges from 0 to 1. A larger winning rate indicates that the radar achieves more successful interactions against the jammer within the evaluation rounds, and therefore reflects stronger radar confrontation capability. Table 4 presents the radar winning rates against jammer models at different capability levels. It can be observed that, as the capability level increases, the proposed method continuously improves the radar’s ability to counter jamming. This demonstrates that the controlled alternating training strategy can effectively enhance radar confrontation performance and reduce the risk of convergence to a local optimum.

To further evaluate the learning capability of the radar agent, the radar network parameters were maintained in a trainable state, enabling the radar policy to evolve continuously during training. The network was randomly initialized, and Monte Carlo Tree Search (MCTS) was integrated into the learning framework to guide policy improvement. In addition, the L1–L7 jammer models obtained in the previous experiments were introduced progressively as opponents. Specifically, jammer models with increasing capability levels were incorporated after 20 k, 40 k, …, and 120 k training episodes, respectively.

**Figure 10:**
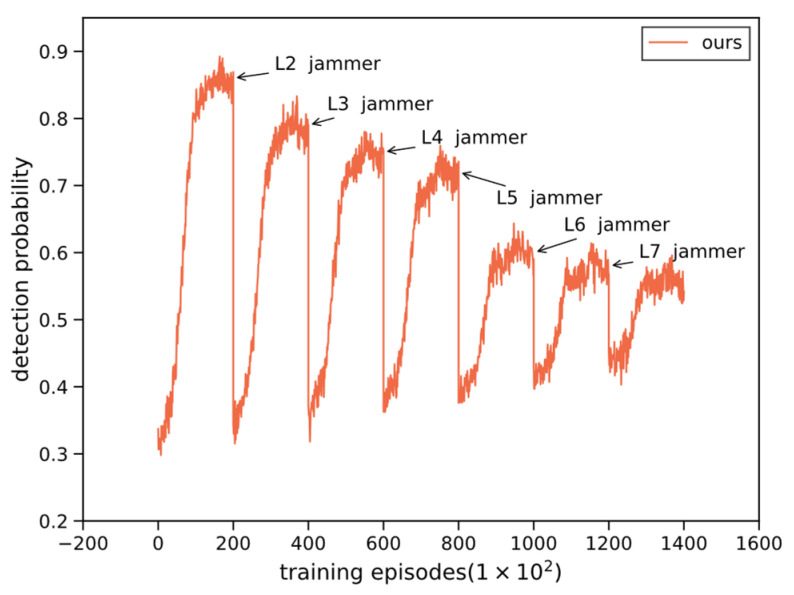
Detection probability of the radar agent against six jammer models of different intelligence levels.

Figure 10 shows the variation in the radar agent’s detection probability when jammer models with different capability levels, from L2 to L7, are introduced progressively during training. Overall, as the capability level of the jammer increases, the peak detection probability of the radar gradually decreases, indicating that higher-level jammer models impose stronger suppression on the radar.

At the beginning of each training stage, the detection probability drops noticeably due to the introduction of a stronger jammer model. As training proceeds, however, the radar agent gradually adapts to the current jamming environment through policy updates, leading to a recovery in detection performance. For example, when confronting the L2 jammer, the detection probability can increase to above approximately 0.85, whereas when confronting the L7 jammer, it remains around 0.55, suggesting that the high-capability jammer significantly increases the difficulty of confrontation.

These results demonstrate that progressively introducing jammer models with different capability levels can establish a curriculum-like training process from easy to difficult, thereby preventing the radar agent from suffering severe performance degradation when directly exposed to a strong jammer at the early training stage. Moreover, the radar agent exhibits a certain degree of recovery and improvement at each stage, confirming that the proposed method can continuously optimize the radar policy in a dynamic adversarial environment and enhance its adaptive anti-jamming capability.

**Figure 11:**
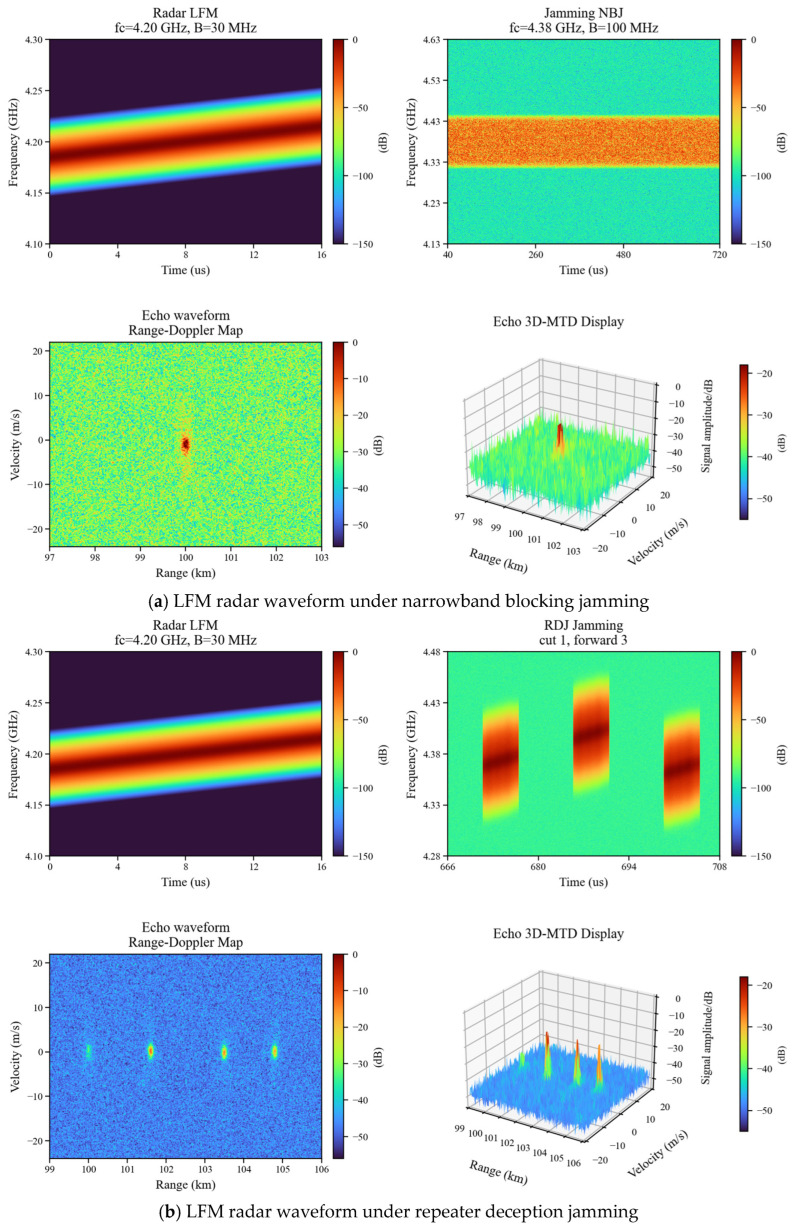

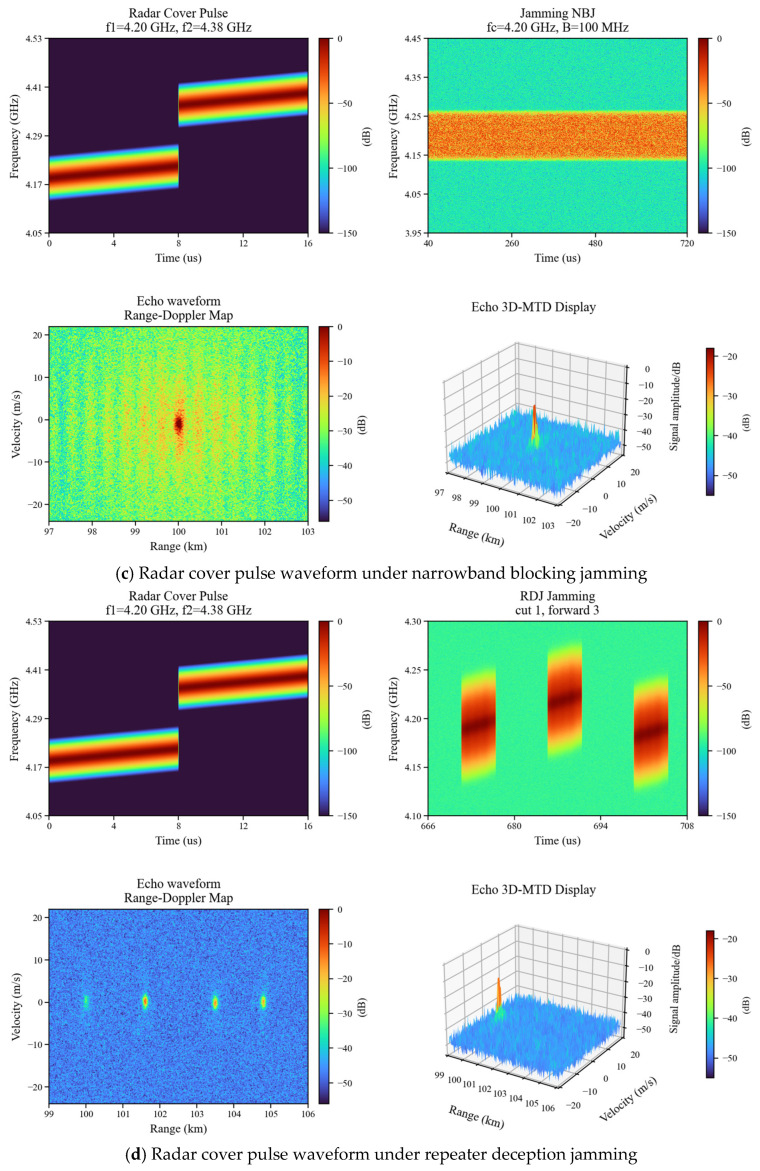
Time-frequency and range-Doppler responses of different radar waveforms under NBJ and RDJ jamming.

The L7 radar model and the L7 jammer model were selected to conduct the radar–jammer confrontation experiment. During the game process, four representative confrontation cases were analyzed: the radar transmits an LFM waveform while the jammer selects NBJ; the radar transmits an LFM waveform while the jammer selects RDJ; the radar transmits a CP waveform while the jammer selects NBJ; and the radar transmits a CP waveform while the jammer selects RDJ. The corresponding results are shown in Figure 11. In each row, the first column presents the time–frequency representation of the radar waveform, the second column shows the time–frequency representation of the jamming waveform, the third column gives the range–Doppler (RD) map of the received echo, and the fourth column displays the three-dimensional moving target detection (3D-MTD) result after filtering and other signal processing operations.

Taking Figure 11d as an example, the radar selects the cover pulse waveform as its anti-jamming waveform. Specifically, the transmitted cover pulse lasts for 20 μs with a frequency offset of −12 MHz, while the detection radar pulse is transmitted from 20 μs to 40 μs with a frequency offset of 12 MHz. The jammer intercepts the component with a frequency offset of −12 MHz and subsequently forwards three deceptive jamming waveforms at the same frequency. As observed from the RD map in Figure 11d, the forwarded deceptive jamming signals have relatively high energy. Without matched filtering, the radar would receive multiple false target responses. However, owing to the advantageous waveform-selection behavior of the radar cover pulse, the MTD result of the target can still be successfully generated after matched filtering. As shown in Figure 11d, the velocity and range information of the target remain clear, demonstrating the effectiveness of the radar anti-jamming decision.

### 5.3. Comparative Evaluation with Multi-Agent Reinforcement Learning Algorithms

**Figure 12:**
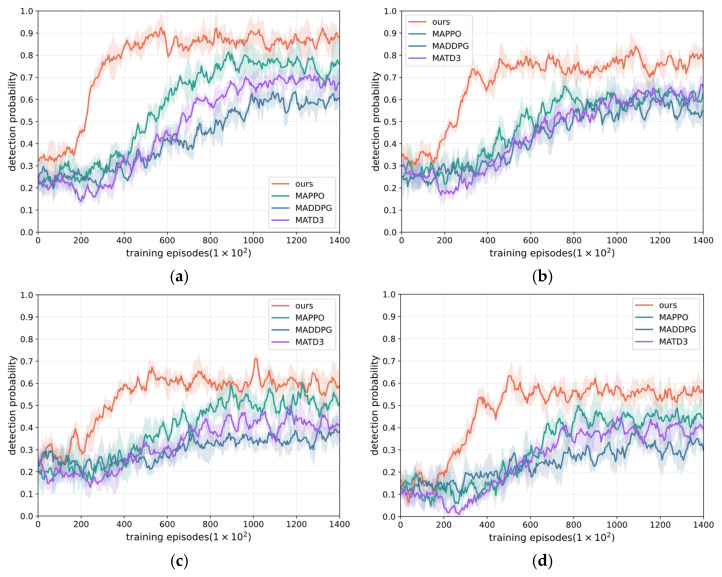
Training performance of the radar agent against jammer models with different capability levels. (**a**) against L1 jammer; (**b**) against L3 jammer; (**c**) against L5 jammer; (**d**) against L7 jammer.

This section evaluates the performance differences between the proposed method and three representative deep reinforcement learning algorithms from the radar perspective. In the experiments, radars equipped with the four intelligent methods were separately tested against L1-, L3-, L5- and L7-level jammers. The detection probability was adopted as the performance metric to assess the anti-jamming capability of the radar. The three deep reinforcement learning algorithms are MADDPG [24], MAPPO [25], and MATD3 [26]. The average radar detection probabilities achieved by the four algorithms are shown in Figure 12. The horizontal axis represents the number of training episodes, while the vertical axis represents the average detection probability of the radar in each episode.

Compared with the three baseline algorithms, the proposed method consistently achieves the highest detection probability and the fastest convergence rate under all jammer capability levels. As shown in Figure 12a–d, the proposed method rapidly improves radar detection performance in the early training stage and reaches a stable policy earlier than the other algorithms. This advantage is mainly attributed to the planning and action-selection capability of MCTS, which enables the radar agent to evaluate potential future radar–jammer interactions before action execution. Therefore, the proposed method can obtain more informative training trajectories and improve sample efficiency in the high-dimensional decision space.

A comparison among Figure 12a–d shows that the performance of all algorithms decreases as the jammer capability increases from L1 to L7. When confronting the L1 jammer, the proposed method converges to a detection probability of approximately 0.86, whereas the value decreases to approximately 0.56 under the L7 jammer. Nevertheless, the proposed method maintains a clear performance advantage over the baselines in all cases, indicating its robustness and adaptability under increasingly strong jamming conditions.

MADDPG shows the slowest learning speed among the compared methods. Although its detection probability gradually increases during training, its final performance remains lower than that of the other algorithms, especially in the L5 and L7 jammer scenarios. This may be attributed to its limited exploration efficiency in high-dimensional state-action spaces and the absence of an explicit planning mechanism, which makes the learned policy more susceptible to local optima.

MAPPO exhibits relatively stable learning behavior and generally performs better than MADDPG. Its detection probability steadily improves as training proceeds, benefiting from the clipped policy update mechanism that stabilizes policy optimization. However, under strong jamming conditions, MAPPO still shows slower convergence and lower final performance than the proposed method, indicating that policy-gradient-based learning alone may be insufficient to fully exploit long-term radar–jammer interaction information in this task.

MATD3 benefits from the twin-critic mechanism and achieves more stable learning than MADDPG by alleviating Q-value overestimation. However, its detection probability remains consistently below that of the proposed method. As observed in the early training stage, MATD3 still exhibits noticeable fluctuations and requires more interactions to learn effective anti-jamming strategies.

To further evaluate the computational complexity and implementation feasibility of the proposed method, a comparative analysis with MADDPG, MAPPO, and MATD3 was conducted in terms of model size and computational cost. As shown in Table 5, all methods were evaluated using the complete dual-agent system under the same precision setting. The proposed radar and jammer policy–value models each contained approximately 4.58 million parameters, resulting in approximately 9.15 million parameters and 36.6 MB of model storage for the complete dual-agent system.

Compared with the baseline algorithms, the proposed method introduces additional computational cost because MCTS repeatedly evaluates candidate actions through the policy–value networks. The forward-computation cost of one complete dual-agent policy–value evaluation is approximately 18.30 MFLOPs. When 100 MCTS simulations are performed during one decision cycle, the corresponding neural-network computation is approximately 1.83 GFLOPs. This value represents the repeated policy–value evaluations and does not include tree traversal, node expansion, radar–jammer state transition, waveform generation, or signal-processing operations.

Although the proposed method requires additional decision-time computation compared with the baseline algorithms, the results in Figure 12 show that it achieves faster convergence and requires fewer training interactions to reach a stable policy. Therefore, the proposed method presents a trade-off between increased search computation and improved sample efficiency and convergence performance. The successful closed-loop execution on the RTX 4090-based semi-physical platform further demonstrates that the computational cost remains acceptable under the current waveform and action spaces.

### 5.4. Performance of the Algorithm on the Semi-Physical Validation Platform

To further evaluate the practical applicability of the proposed method, the trained radar and jammer agents were deployed on the semi-physical platform described in Section 4. Compared with the simulation environment, the semi-physical platform involves real signal generation, acquisition, processing, and closed-loop control, thereby providing a more realistic validation environment for intelligent radar–jammer confrontation.

**Figure 13:**
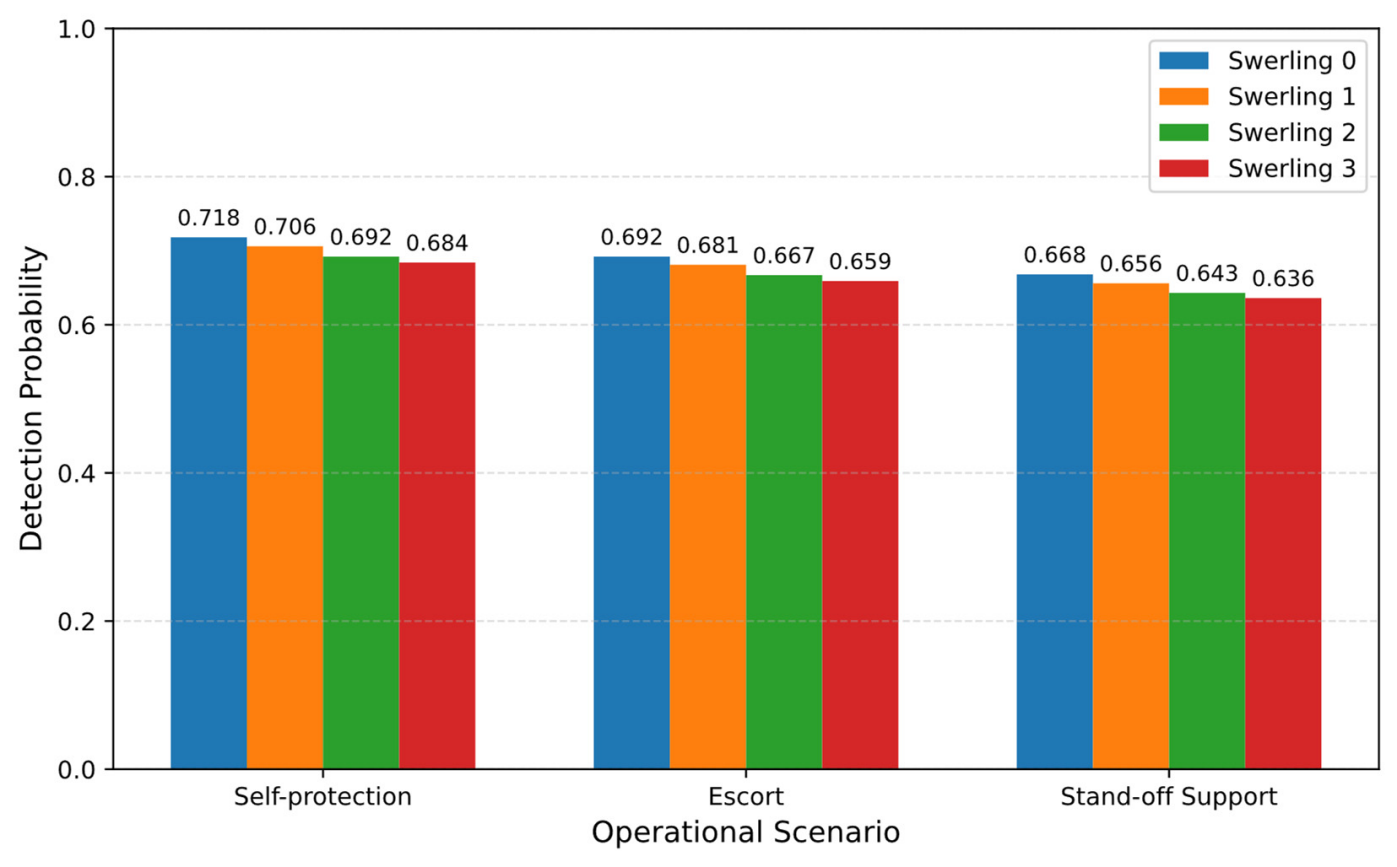
Detection probability of the proposed radar under different operational scenarios and target fluctuation models.

The platform supports three operational scenarios, namely self-protection jamming, escort jamming, and stand-off support jamming. It also supports four target fluctuation models, including Swerling 0, Swerling 1, Swerling 2, and Swerling 3. Based on these platform configurations, this section evaluates the effectiveness and robustness of the proposed method under different operational scenarios and target RCS fluctuation characteristics. In this experiment, the trained L7 radar model and L5 jammer model were deployed on the semi-physical validation platform. The detection probabilities under different operational scenarios and target fluctuation models are shown in Figure 13.

As shown in Figure 13, the proposed method maintains stable anti-jamming performance under different operational scenarios and target fluctuation models on the semi-physical platform. The detection probability is highest in the self-protection scenario, followed by the escort and stand-off support scenarios, which is consistent with the increasing confrontation complexity. For each scenario, the detection probability gradually decreases from Swerling 0 to Swerling 3 due to stronger target echo fluctuations. Nevertheless, all results remain above 0.63, demonstrating the robustness of the proposed method to both scenario variations and target RCS fluctuations.

**Figure 14:**
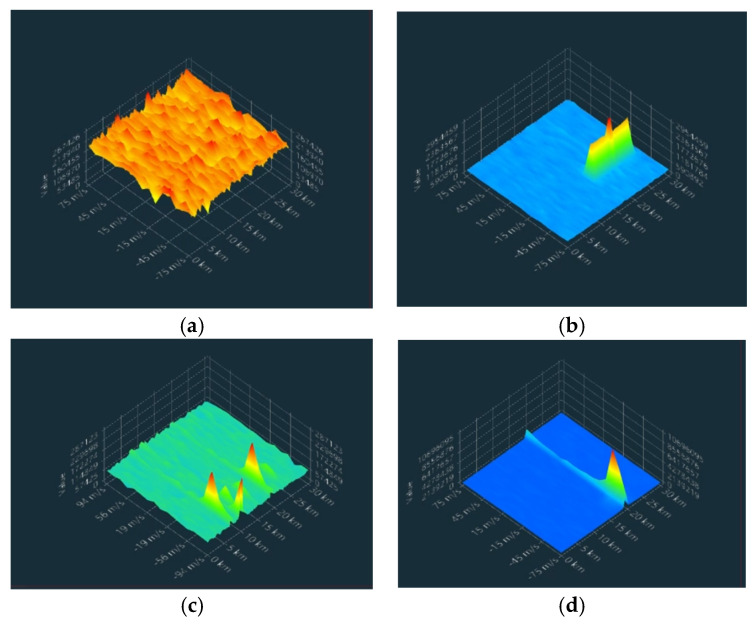
Measured range–Doppler maps obtained from the semi-physical platform under different jamming and anti-jamming conditions: (**a**) target echo under narrowband blocking jamming; (**b**) recovered target echo after applying the anti-NBJ strategy; (**c**) target echo and false-target echoes under repeater deception jamming; (**d**) recovered target echo after applying the anti-RDJ strategy.

**Figure 15:**
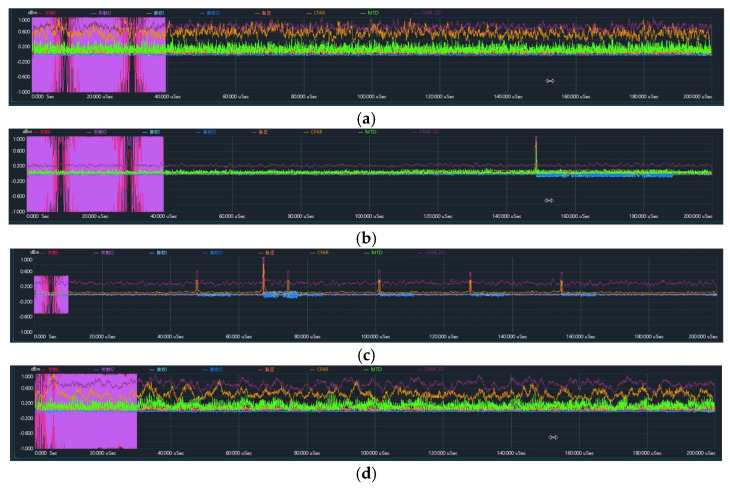
Measured time-domain waveforms obtained from the semi-physical platform under different jamming and anti-jamming conditions: (**a**) received waveform under narrowband blocking jamming; (**b**) waveform after applying the anti-NBJ strategy; (**c**) received waveform under repeater deception jamming; and (**d**) waveform after applying the anti-RDJ strategy.

During the experiment, the platform recorded the range–Doppler (RD) maps and the corresponding time-domain waveforms under different jamming conditions to verify the effectiveness of the proposed method at the signal level. Figure 14 shows the RD maps before and after anti-jamming processing under narrowband jamming (NBJ) and repeater deception jamming (RDJ), while Figure 15 presents the corresponding measured waveform results.

As shown in Figure 14a, narrowband blocking jamming raises the background energy over a wide region of the range–Doppler plane, making the target echo difficult to distinguish. After the anti-NBJ strategy is applied, the interference energy is significantly reduced and the target echo is recovered, as shown in Figure 14b. Under repeater deception jamming, multiple false-target echoes appear in the range–Doppler plane, as shown in Figure 14c. After the anti-RDJ strategy is applied, the false-target echoes are weakened and the true target echo becomes dominant, as shown in Figure 14d. These measured results demonstrate that the proposed method can adaptively select effective waveform strategies against both suppression and deception jamming on the semi-physical platform.

As shown in Figure 15a, the received waveform under narrowband blocking jamming contains strong interference components and pronounced amplitude fluctuations. After the anti-NBJ strategy is applied, the interference component is significantly reduced and the target echo becomes more distinguishable, as shown in Figure 15b. Under repeater deception jamming, multiple delayed and repeated waveform components appear in the received signal, as shown in Figure 15c. After the anti-RDJ strategy is applied, these deceptive components are weakened and the waveform associated with the true target echo becomes more prominent, as shown in Figure 15d. These results confirm that the radar and jammer modules on the semi-physical platform can complete closed-loop signal interaction according to the decisions of the intelligent agents. They also demonstrate that the radar agent can adaptively select effective waveform strategies to suppress or avoid different types of jamming. Therefore, the semi-physical validation results verify the feasibility and effectiveness of the proposed method in practical radar–jammer confrontation scenarios.

To further evaluate the real-time performance of the proposed method on the HIL platform, the execution latency was measured under different MCTS search configurations. The total decision latency consists of the policy-value network inference time and the MCTS search time. As shown in Table 6, the network inference latency remains nearly unchanged because the network architecture is fixed, whereas the MCTS search latency increases with both the tree depth D and the number of simulations M. Under the default configuration of D=5 and M=100, corresponding to an additional search computation of approximately 1.83 GFLOPs, the total decision latency is 29.0 ms. This result indicates that the proposed method can complete strategy computation within the closed-loop strategy-update interval of the current HIL platform.

These results demonstrate that the computational overhead of MCTS can be adjusted through the search depth and simulation budget, providing a practical trade-off between decision quality and execution efficiency.

The scalability of MCTS is also affected by the size of the action space. An expanded action space increases the branching factor of the search tree and reduces the search coverage of individual actions under a fixed simulation budget. Although the proposed MCTS selectively expands high-value branches rather than exhaustively traversing the complete tree, a substantially larger action space may still require more simulations and self-play samples to maintain comparable decision quality, thereby increasing convergence time and hardware computational overhead. Therefore, the current action space represents a trade-off between waveform decision granularity and practical computational feasibility. Further expansion of the action space may require adaptive simulation budgets, hierarchical action decomposition, or progressive widening to maintain efficient search.

## 6. Conclusions

This paper proposed an intelligent jammer-assisted radar anti-jamming evolution method based on the AlphaZero framework. The waveform-level confrontation between the radar and jammer was formulated as a two-agent sequential decision-making game, in which the radar progressively improves its anti-jamming policy through dynamic interactions with intelligent jammer models. By integrating self-play learning with Monte Carlo Tree Search, the proposed method improves interaction-sample quality, action evaluation, and training efficiency. Simulation results demonstrate faster policy convergence and improved radar detection performance under different jammer capability levels. In addition, a semi-physical hardware-in-the-loop platform was developed to evaluate practical feasibility. The results show that the radar agent can adaptively select effective waveform strategies under different jamming conditions, supporting the potential of search-guided dual-agent learning for intelligent radar anti-jamming.

The main limitation of the current framework is the scalability of MCTS, as the computational cost increases rapidly with larger waveform and jamming action spaces. In addition, the current experiments consider representative action types and do not fully reproduce all uncertainties of real field deployment. Future work will focus on improving search efficiency and extending the framework to richer waveform libraries and more realistic confrontation environments.

## Figures and Tables

**Table 1:** Parameters used in training the proposed models.

Agent	Waveform Type	Parameter Type	Notation	Value
Radar	LFM	transmit frequency	$f_{t}^{R}$	3–5 GHz
		transmit bandwidth	$B_{t}^{R}$	10–50 MHz
	CP	cover-pulse frequency	$f_{t,c}^{R}$	3–5 GHz
		detection-pulse frequency	$f_{t,d}^{R}$	3–5 GHz
		cover-pulse width	$T_{t,c}^{R}$	10–50 μs
		detection-pulse width	$T_{t,d}^{R}$	10–50 μs
Jammer	NBJ	transmit frequency	$f_{t}^{J}$	3–5 GHz
		transmit bandwidth	$B_{t}^{J}$	10–400 MHz
	RDJ	intercept time	$T_{t,rec}^{J}$	10–50 μs
		repetition number	$N_{t,rep}^{J}$	1–5

**Table 2:** Hyperparameters settings used for agent simulation.

Agent	Parameter Type	Notation	Value
Radar	radar transmission power	$P_{r}$	30 kW
	radar antenna gain	$G_{r}$	32 dB
	target range	R	100 km
	false alarm rate	$r_{false}$	10^−4^
Jammer	jammer transmission power	$P_{j}$	1 kW
	jammer antenna gain	$G_{j}$	10 dB

**Table 3:** Network architecture and parameter settings of the radar and jammer agents.

Parameter Design	Radar Agent	Jammer Agent
Observation dimension	128	128
Hidden-layer dimensions	1024–2048–1024	1024–2048–1024
Activation function	ReLU	ReLU
Value-head dimension	240–1	240–1
Replay buffer	10,000	10,000
Mini-batch size	256	256
Learning rate	1 × 10^−4^	1 × 10^−4^
Discount factor	0.99	0.99
MCTS simulations	100	100
$c_{puct}$	1.5	1.5

**Table 4:** Radar winning rates against jammers with different capability levels.

	L1 Jammer	L2 Jammer	L3 Jammer	L4 Jammer	L5 Jammer	L6 Jammer	L7 Jammer
L1 radar	0.56	0.45	0.37	0.30	0.24	0.18	0.13
L2 radar	0.64	0.54	0.47	0.39	0.31	0.25	0.19
L3 radar	0.71	0.62	0.55	0.46	0.38	0.32	0.26
L4 radar	0.78	0.69	0.61	0.56	0.48	0.40	0.33
L5 radar	0.84	0.76	0.68	0.60	0.54	0.46	0.39
L6 radar	0.89	0.82	0.74	0.67	0.61	0.55	0.48
L7 radar	0.92	0.86	0.80	0.73	0.66	0.59	0.56

**Table 5:** Estimated computational cost of the proposed dual-agent system.

Method	Model Parameters	Model Storage	Forward Computation	Additional Search Computation
MADDPG	≈6.12 M	≈24.5 MB	≈12.24 MFLOPs	\
MAPPO	≈7.36 M	≈29.4 MB	≈14.72 MFLOPs	\
MATD3	≈9.18 M	≈36.7 MB	≈18.36 MFLOPs	\
Ours	≈9.15 M	≈36.6 MB	≈18.30 MFLOPs	≈1.83 GFLOPs

**Table 6:** Execution latency of the proposed method under different MCTS configurations.

Tree Depth D	Simulations M	Network Inference (ms)	MCTS Search (ms)	Total Decision Latency (ms)
3	25	2.6	4.8	7.6
	50	2.6	8.9	11.8
	75	2.7	13.1	16.1
	100	2.7	17.4	20.4
5	25	2.6	7.1	10.0
	50	2.7	13.5	16.5
	75	2.7	19.8	22.9
	100	2.8	26.2	29.4

## Data Availability

Dataset available on request from the authors.
